# Topochemical control in desolvation of coordination polymers

**DOI:** 10.1107/S2052252515003395

**Published:** 2015-02-26

**Authors:** Matteo Lusi

**Affiliations:** a Chemical and Environmental Science, University of Limerick, Limerick, Ireland

**Keywords:** coordination polymers, topochemical control, solid–gas reactions

## Abstract

Reactions in the solid state are at the core of crystal engineering as they can result in new crystalline phases that are not always accessible by traditional solution methods. The work of Brammer and co-workers [Wright* et al*. (2015), *IUCrJ*, **2**, 188–197] represents a clear example of this potential as applied to the synthesis of a silver–phenazine coordination polymer.

In the past fifty years, one of the major challenges for crystallographers and *crystal engineers* has been the rationalization and exploitation of the molecular pre-aggregation for reactions that occur in the solid state. Indeed, one of the first appearances of the term ‘crystal engineering’ coincides with Schmidt’s investigation of the 2+2 photochemical cycloaddition in the polymorphs of *cis*-cinammic acid (Schmidt, 1971 ▶). That experiment showed that ‘freezing’ molecules in the appropriate fashion is the key for some reactions to occur, and that the crystalline state can serve as a template for those reactions that depend on the reciprocal molecular orientation (topochemical control). A great deal of research on molecular crystals involves reactions within solids, triggered by photo or thermal events (Toda, 2005 ▶), or at the interface of the solid (James *et al.*, 2012 ▶) with other solids and molten phases. These reactions can be difficult to identify by standard diffraction experiment and require *ad hoc* crystallographic analysis (Lusi & Barbour, 2013 ▶; Poulain *et al.*, 2014 ▶; Casaretto *et al.*, 2015 ▶).

Solid–gas reactions have received particular attention owing to their potential exploitation in chemical sensing, storage and separation (Perry *et al.*, 2009 ▶). Although most of them consist of physisorption of inert guests onto the internal surface of a porous material, some remarkable examples of host–guest chemistry describe molecular diffusion of volatile species within close-packed crystals (Atwood *et al.*, 2002 ▶). In molecular crystals, intricate equilibria are known to exist between the pure compounds and their hydrogen-bonded water and alcohol solvates (Braga *et al.*, 2008 ▶; Braun *et al.*, 2012 ▶). Similarly, ammonia and hydrohalic acids can reversibly coordinate to metals in discrete (Mínguez Espallargas *et al.*, 2007 ▶) and polymeric complexes (Adams *et al.*, 2007 ▶), while clathration of aromatic species can be driven by the increased lattice energy deriving from weak van der Waals interactions (Lusi & Barbour, 2012 ▶). Even the simple selective exposure of a given crystallographic face to water vapour can trigger molecular reorientation (Jiang *et al.*, 2013 ▶). These phenomena require dramatic structural reorganization (Caira & Nassimbeni, 1996 ▶), which contrasts with the rigidity typically associated with the crystalline state.

Now Brammer and co-workers (Wright* et al*., 2015 ▶) explore this idea further by investigating a family of silver–phenazine coordination polymers: {[Ag_4_(O_2_C(CF_2_)_2_­CF_3_)_2_(phenazine)_2_(arene)_*k*_]·*J*(arene)}_*n*_ (arene = toluene, xylene). In these compounds the aromatic solvent has a double function: as a ligand it is coordinated to open metal sites and as a guest it is included within the cavities present in the structures. It follows that the structure and composition of the product depend on the solvent used in the synthesis. Besides a novel arene–silver coordination geometry, the paper reports on the solid-state reactivity of the family of compounds. The authors observe that volatile solvent can diffuse out of the non-porous crystal causing major structural transformation, which results in a mixture of products. In total, three crystalline phases were identified: two have been characterized as polymorphs – perhaps polytypes (Feller & Cheetham, 2008 ▶) or supramolecular isomers (Moulton & Zaworotko, 2001 ▶) – of the coordination polymer {[Ag_4_(O_2_C(CF_2_)_2_CF_3_)_2_(phenazine)_2_]}_*n*_, while the third remains unknown.

Solvent removal can be achieved by heating or exposing the sample to air, but also by exposing the solvated microcrystalline powder to the vapour of alcohols and arenes (other than those already included in the structure). Under the attempted experimental conditions, guest substitution was observed only between toluene and *p*-xylene, and even in that case it was only partial. Most importantly, the relative amount of the phases produced depends on both the structural features of the reacted material and the method used to eliminate the solvent. A sound structural analysis relates those observations with the topochemical character of the reactions. Remarkably, one of the desolvated forms could not be isolated by wet methods. A single crystal was instead isolated from the thermal elimination experiment. Since the reactions afford crystalline particles that are large enough for single-crystal diffraction it can be imagined that the molecular reorganization is concerted or that there is a certain degree of flexibility in the polymeric chains.

An archaic interpretation of the solid physical state portrays crystals as rigid assemblies of atoms and molecules that are buried into fixed positions from which they cannot move (Dunitz *et al.*, 1988 ▶). This view is countered by many examples of solid-state reactions that occur with considerable molecular reorganization. The work of Brammer and co-workers is another such example. It shows that novel coordination polymers can be produced from solvated precursors, the product depending on the molecular arrangement in the reacting structure as well as the conditions used to eliminate the guests. Given the wide potential applications of coordination polymers (as porous or conducting or magnetic materials), all the synthetic strategies shall be considered, which might afford the desired architecture. In this sense, solvent elimination represents a powerful tool to access crystalline products under topochemical control for the rational design of novel materials.
